# User experiences of patients with post-acute COVID-19 syndrome receiving occupational therapy telerehabilitation

**DOI:** 10.3389/fnhum.2025.1551631

**Published:** 2025-04-25

**Authors:** Lynn H. J. Kok, Jenny T. Gu, Jessica T. Y. Kung, Shera S. Liang, Pablo Cruz Gonzalez, Fong Mei Toh, Emily Sin, Kenneth N. K. Fong

**Affiliations:** ^1^Department of Rehabilitation Sciences, The Hong Kong Polytechnic University, Hong Kong, Hong Kong SAR, China; ^2^Rehabilitation Research Institute of Singapore (RRIS), Nanyang Technological University (NTU), Singapore, Singapore; ^3^Singapore Institute of Technology, Singapore, Singapore; ^4^School of Design, The Hong Kong Polytechnic University, Hong Kong, Hong Kong SAR, China; ^5^Research Centre for Assistive Technology, The Hong Kong Polytechnic University, Hong Kong, Hong Kong SAR, China

**Keywords:** post-acute COVID-19 syndrome, telerehabilitation, occupational therapy, usability, mixed-methods study, long COVID

## Abstract

**Background:**

Patients with post-acute COVID-19 syndrome, also referred to as “long COVID,” may face persistent physical, cognitive and psychosocial symptoms which can be challenging to manage given the strict social distancing measures imposed during the pandemic. Telerehabilitation (TR) became increasingly common during COVID-19 pandemic and has been applied to post-acute COVID-19 conditions in previous clinical studies, and it was reported that patients’ symptoms were alleviated and their overall health improved. This study examined the usability and acceptability of TR by occupational therapists delivered for patients suffering from post-acute COVID-19 in Hong Kong.

**Methods:**

In this mixed-methods usability study, participants rated items on the System Usability Scale (SUS) and completed a semi-structured questionnaire via audio-recorded telephone calls. Descriptive data were used to summarize the quantitative data, and thematic analysis was applied to analyze the qualitative data.

**Results:**

Twelve participants (mean age 56.5 years) who had completed a 6-week TR program via the Caspar Health system were recruited for the study. A median SUS score of 56.25 was reported for its usability, despite 83% of the participants viewed the TR system as fairly acceptable. Four themes, namely perception of using the TR system - performance expectancy of TR, other psychosocial and environmental factors, and intention to use TR, were generated on the basis of the participants’ interviews. Most participants reported their willingness to continue using TR and that they would recommend it to other patients.

**Conclusion:**

Most of the participants were receptive to TR and perceived health benefits from its use. Future research could consider integrating the perspectives of both occupational therapists and patients to generate a more comprehensive understanding of the facilitators of and the barriers to TR for patients who experience long COVID.

## Introduction

As the coronavirus disease 2019 (COVID-19) pandemic stretches into its third year, it continues to bring devastating effects to various economies and communities with the emergence of highly contagious variant strains (Gong et al., 2022). Many cities have fought the dynamic pandemic wave patterns with strict social distancing measures since mid-2020 (Kwok et al., 2020). These measures have included the suspension of onsite classes and the closure of public facilities at the height of the recent COVID-19 wave to curb the spread of transmission (Cacciapaglia et al., 2021; GovHK, 2022; Tang, 2022).

A meta-analysis by Chen et al. (2022) reported that approximately 40% of patients globally continue to suffer from post-acute COVID-19 syndrome (PACS), also referred to as “long COVID,” a condition characterized by persistent and myriad symptoms experienced by patients 3 months after the onset of disease and these symptoms may last for at least 2 months (Lopez-Leon et al., 2021; Marshall, 2021; Nalbandian et al., 2021; Tabacof et al., 2022; World Health Organization, 2022). The prevalence of long COVID is approximately 10–20% of the people infected with COVID (Corononavirus.gov.hk, 2022). In the US, one in five adults who had had COVID have experienced long COVID 3 months later whereas the estimate was at one in 10 in the UK (Sutherland, 2023). As the COVID-19 pathogen targets multiple organs in the body, the common physical symptoms of long COVID range from fatigue and dyspnea to psychological and cognitive symptoms, including cognitive disturbances (also known as “brain fog”), depression, and anxiety (Nalbandian et al., 2021; Proal and VanElzakker, 2021; NHS, 2022). Researchers have found that the COVID virus can reach the brain through the olfactory bulb connecting to other brain areas causing the cognitive disturbances and that the fatigue is often affiliated to a heart condition called postural orthostatic tachycardia syndrome (Sutherland, 2023). Moreover, evidence also shows that some face significant psychological distress—anxiety, depression, and post-traumatic stress—as a result of prolonged hospitalization and social isolation (Bonazza et al., 2021; Srivastava et al., 2021; Toulabi et al., 2021). Face-to-face conventional rehabilitation services have been suspended during the pandemic waves, and limited resources have made it difficult for patients to manage PACS (Lau et al., 2021). While it is uncertain how long PACS may last for different individuals, the persistent symptoms are negatively associated with their quality of life and social participation (Malik et al., 2022; Montani et al., 2022; Tabacof et al., 2022). Management of PACS involves interdisciplinary medical team because of its heterogeneous symptom manifestations.

Telerehabilitation (TR) uses information and communication technology to provide rehabilitation services remotely (Fong and Kwan, 2020). It became increasingly common during the COVID-19 pandemic as a cost-effective and convenient option for providing rehabilitation services for patients under social distancing measures (Bitar and Alismail, 2021; Kissi et al., 2020; Ku et al., 2021; Tanguay et al., 2021). Also, it offers an accessible route to deliver timely interventions to prevent further deconditioning of patients suffering from residual symptoms post-hospitalization (Chan et al., 2016; Leite et al., 2021; Tanguay et al., 2021). During the COVID-19 pandemic, evidence has shown that TR alleviates the physical symptoms that post-acute COVID-19 patients face and increases their motivation to exercise (Bernal-Utrera et al., 2021; Milani et al., 2021; Li et al., 2021; Wong et al., 2022). Furthermore, these timely TR interventions prevent the worsening of their mental health and improve their overall health (Bernal-Utrera et al., 2021; Li et al., 2021; Vieira et al., 2022). It has allowed healthcare professionals to overcome short-term service disruptions and offer a home-based service to assist Hong Kong residents to cope with anxiety and depression during the COVID-19 pandemic (Choi et al., 2020).

Even though TR is a long-term and affordable solution to deliver rehabilitation services, there are common barriers to its use, such as the lack of technological infrastructure and the difficulty in using new mobile apps reported in previous studies (Arzani et al., 2021; Bernal-Utrera et al., 2021; Werneke et al., 2021). These barriers lower the acceptance of TR among post-acute COVID-19 patients. While most studies have examined the usability of TR among patients with COVID-19, the perceived acceptability of TR has rarely been reported (Bernal-Utrera et al., 2021). Hence, to better understand the factors that influence the use of TR, the user perspectives of post-acute COVID-19 patients need to be examined further (Buabbas et al., 2022; Leite et al., 2021; Werneke et al., 2021). The Caspar Health system is a commercial TR platform developed in Germany that is embedded with a written Chinese and spoken Cantonese program for use in Hong Kong. A previous study showed that older adults who underwent post-hip fracture surgery made significant improvements in falls efficacy (i.e., fall reduction) after receiving TR through the Caspar Health system integrated with smartphones (Li et al., 2022).

The World Federation of Occupational Therapists (WFOT) has acknowledged that TR is an appropriate service delivery model for occupational therapy services (World Federation of Occupational Therapists (WFOT), 2014). Therefore, studying the TR experiences of this patient group would provide deeper insights on the factors that influence their utilization of TR. This study aimed to examine the usability and acceptability of TR by occupational therapists delivered via the digital platform powered by the Caspar Health system in response to the among patients with post-acute COVID-19 syndrome (PACS) in Hong Kong. In this study, the ‘usability’ of the Caspar Health system in providing TR for patients with long COVID was examined in three domains—effectiveness, efficiency, and satisfaction (Brooke, 2013)—and the acceptability of the system was assessed on the basis of the users’ attitudes toward TR and their perceptions of the usefulness of, and their intention to use, the Caspar Health system (Nadal et al., 2020).

## Materials and methods

### Research design

This study explored the experiences of post-acute COVID-19 patients who received a 6-week TR program via the Caspar Health system during the period from November 2021 to February 2022. The study adopted a mixed-methods sequential explanatory procedure that consisted of a quantitative followed by a qualitative phase (Ivankova et al., 2006). The study was conducted by the Department of Rehabilitation Sciences and was approved by the Ethics Committee of the Hong Kong Polytechnic University (Reference Number: HSEARS20220308003).

### Participants

Thirteen post-acute COVID-19 patients who had received TR were recruited by convenience sampling 6 months after the interventions had been completed. The participants were screened for eligibility on the basis of the following criteria: (1) COVID-19 survivors who were 18 years old and above, (2) completed 6 weeks of online-based TR via the Caspar Health app, and (3) no difficulties in verbal communication. Participants were excluded from the study if they did not complete the prescribed TR or possessed other medical conditions that impeded their ability to communicate. Twelve of the original 13 participants were finally enrolled in this study; one was excluded as she did not complete the whole 6-week TR program.

### Treatment program

Participants underwent thorough 6-week program of online-based TR via Caspar Health training system with on loaned iPhones (Scheer, 2021). The Caspar Health e-system is a digital rehabilitation app designed by TechCrunch Disrupt (Caspar Health, Berlin, Germany) that has a traditional Chinese program with Cantonese dialect speech in Hong Kong that was adopted for the native Cantonese speakers in this study.^1^ The Caspar Health TR system has the following features: (1) Therapists set a tailor-made TR program for each patient through the TR system calendar, and customized intervention, such as training videos and frequency, are transferred to the patient’s iPhone through the Caspar Health App; (2) The patient performs the home-based training using the videos, pictures, written and verbal instructions shown on the App; (3) After practice, the patient may upload their training video or verbal feedback to the occupational therapists so that the therapists can update the home program according to the patient’s progress; and (4) Therapists can review patients’ attendance records and communicate with them if needed (Li et al., 2022). Participants with PACS followed a customized TR program consisting of exercise, relaxation, and lifestyle training videos with closed captions scheduled through their e-system calendar programmed by occupational therapists. Five to six specific strengthening and cardiopulmonary exercises at indicated repetitions were prescribed according to their physical performances at the start of their respective TR programs. Relaxation, mindfulness, and energy conservation education videos that aimed to alleviate PACS were also prescribed for their viewing throughout the 6-week TR program. As the patients’ data were transferred and stored in an encrypted cloud in real time, therapists were able to track the compliance rate and communicate with the patients directly if required.

### Outcome measures

Quantitative data were collected from the participants through their responses in the Chinese version of the 10-item System Usability Scale (SUS) (Wang et al., 2019). SUS was used to measure the usability of the Caspar Health system, participants rating their level of agreement on a 5-point Likert scale ranging from 1 (fully disagree) to 5 (fully agree) (Lewis, 2018; Peres et al., 2013). SUS has a cut-off score of 68, a score above 68 being considered above average in its usability of the TR system (U.S. General Services Administration, 2022). SUS scores can also be used to determine the perceived acceptability of TR by sorting them according to the acceptability ranges (Bangor et al., 2008). Individual semi-structured interviews were then conducted to examine the qualitative experience of participants in using the Caspar Health system. The research team first reviewed the semi-structured questionnaires of studies that had examined the attitudes and perspectives of patients toward TR during the COVID-19 pandemic (Aliaga-Castillo et al., 2022; Bernal-Utrera et al., 2021; Maeir et al., 2022) and then designed a distinct set of semi-structured interview questions based on the guidelines for qualitative research in healthcare produced by DeJonckheere and Vaughn (2019) and Pope et al. (2000). The interview questions (see Appendix) were reviewed and amended by an expert panel that consisted of two academic faculty staff in occupational therapy and an occupational therapist with extensive experience of using TR.

### Procedures

A research team member scheduled and conducted the individual interviews via audio-recorded phone calls. Each participant was provided with the study information sheet, and the purpose of the study was verbally explained to them. Verbal informed consent to participate in the study was collected by audio recordings before the interviews. The participants could voluntarily withdraw at any time. Each interview lasted for approximately 15–30 min. To ensure the credibility of the data collected, the interviews were supervised by a faculty staff member who was not part of the research team. The interviews were then transcribed verbatim into traditional Chinese by the same interviewer. All of the data were kept confidential and stored in an encrypted cloud server that was only accessible by the research team.

### Data analysis

Each participant’s SUS score was calculated as a percentage score on the basis of the SUS protocol (U.S. General Services Administration, 2022). The scores were then sorted into three levels—acceptable, marginally acceptable, and not acceptable—reflecting the participants’ perceived acceptance of the use of the Caspar Health system (Bangor et al., 2008). Descriptive statistics were used to summarize the demographic characteristics of the participants. The thematic analysis framework by Braun and Clarke (2006) was applied to analyze the transcripts to generate the codes and themes. To ensure the trustworthiness and rigor of the data, strategies such as investigator triangulation and audit by external researchers were used. Each interview was analyzed by three researchers individually first, before team meetings were held to compare their analyses. Possible themes were explored thereafter, and the data were triangulated with the fieldnotes from the interviewer before translating the themes into English. An audit was conducted by the expert panel which oversaw the research but was not involved in the data collection.

## Results

Twelve participants, four males and eight females with a mean age of 56.5 years (range 25–72), were recruited for this study; 33.3% (*n* = 4) of the participants had attained secondary school diplomas and 66.7% (*n* = 8) had university degrees. Details of the participants can be found in Table 1.

**TABLE 1:** Demographic information.

Participant	Age	Gender	Education	SUS scores
1	62	Female	Secondary School Diploma	55
2	49	Male	University	62.5
3	25	Female	University	77.5
4	72	Female	Secondary School Diploma	57.5
5	57	Female	Secondary School Diploma	17.5
6	53	Male	Secondary School Diploma	70
7	62	Female	University	55
8	53	Female	Secondary School Diploma	67.5
9	38	Male	University	55
10	72	Male	Secondary School Diploma	55
11	65	Female	Secondary School Diploma	32.5
12	70	Female	Secondary School Diploma	72.5

The SUS scores of the 12 participants ranged from 17.5 to 77.5, with a mean of 56.5 (S.D. 16.9) and a median of 56.25; these results fell below the expected cut-off score of 68 in its usability. Table 2 displays the participants’ level of acceptability as defined by Bangor et al. (2008) through their SUS scores. As shown in Table 2, 83% (*n* = 10) of the participants viewed the TR system to be marginally acceptable or acceptable, while participants 5 and 11 rated the Caspar Health system as not acceptable. The mean score for each item in the SUS ranged from 2.5 to 3.6 as most participants gave a rating of 3 (Neutral) in their responses, which are presented in Table 3.

**TABLE 2:** Level of acceptability of TR system.

Level of acceptability (Bangor et al., 2008)	Range of SUS scores	Percentage of participants
Acceptable	70–100	25%
Marginally acceptable	50–70	58%
Not acceptable	Below 50	17%

**TABLE 3:** Mean score of each item in SUS.

ID	Item	Mean score
1	I think that I would like to use this system frequently	3.5
2	I found the system unnecessarily complex	2.8
3	I thought the system was easy to use	3.2
4	I think that I would need the support of a technical person to be able to use this system	3
5	I found the various functions in this system were well integrated	3.6
6	I thought there was too much inconsistency in this system	3
7	I would imagine that most people would learn to use this system very quickly	3.6
8	I found the system very cumbersome to use	2.5
9	I felt very confident using the system	3
10	I needed to learn a lot of things before I could get going with this system	3

**FIGURE 1:**
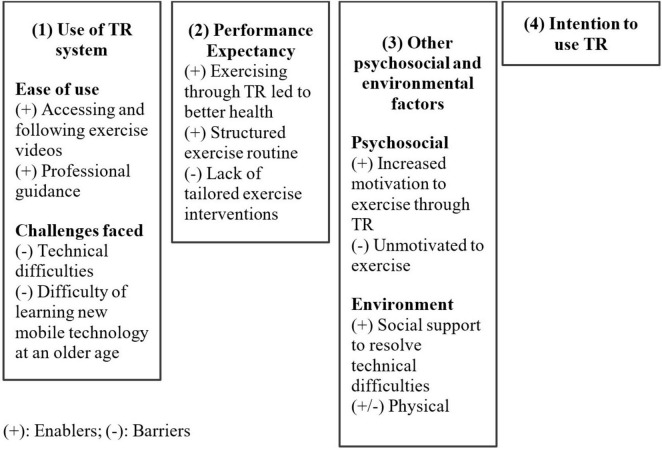
Summary of themes and their subthemes.

The qualitative data analysis found 94 codes and four main themes. The themes were (1) perception of using the Caspar Health system, (2) performance expectancy of TR, (3) other psychosocial and environmental factors, and (4) intention to use TR. The themes and their subthemes are summarized in Figure 1.

***Theme 1 - Use of TR system***

From the participants’ responses on their experiences of using the Caspar Health system, two subthemes emerged to support the ease of using the TR system, while two subthemes highlighted the challenges faced. The two subthemes that described the ease of use were accessing and following exercise videos and professional guidance.

**1) Accessing and following exercise videos**

Nine participants used terms such as “*simple, smooth process, convenient and easy*” to describe the process involved in accessing their e-calendars and viewing their individualized lists of exercises. Participants further shared that as the exercises were presented in video format with subtitles and verbal instructions, they were able to follow and perform the repetitions of the exercises with ease. Participant 3 commented that “*because it had videos to follow,*” it was easy to use the TR system, adding that “*it would be hard to follow if the exercises only consisted of words*”.

**2) Professional guidance**

Some participants perceived the TR system as a reliable app where the exercises were guided by healthcare professionals and specifically described it as being “*authentic and professional*”. Participant 1 highlighted that she felt there were “*no significant differences between Caspar Health and face-to-face supervision from therapists*” and that she could perform the exercises without the need for a therapist.

Two subthemes that described the challenges that the participants faced during their use of TR were technical difficulties and difficulty of learning new mobile technology at an older age.

**3) Technical difficulties**

Most of the technical issues that five participants reported were difficulties with logging onto the system, application hangs, and the inability to view the exercise videos after a system update. They reported that they were first-time iPhone users and hence faced significant challenges in resolving the issues faced using a loaned and unfamiliar mobile phone. Some went on to seek assistance from the study team to resolve the technical issues they encountered in order to continue with the intervention.

**4) Difficulty of learning new mobile technology at an older age**

With the average age of the participants being 56.5 years, some elderly participants shared they were unable to recall and learn the steps to use unfamiliar technology as they got older. Some used the terms like “*very complicated, difficult and troublesome*” to describe their unpleasant experiences. Other participants mentioned that although the study team was helpful in teaching them the steps repeatedly, they preferred face-to-face therapy to avoid the hassle of navigating through the app. Participant 4 shared their thoughts: “*because we are older, it is not easy to remember*… *you need to demonstrate it to me then I can perform it*”.

***Theme 2 - Performance expectancy***

In this study, the concept of performance expectancy relates to how refers to how individuals perceive that TR would assist them to achieve health-related outcomes (Venkatesh et al., 2003). Two enabling subthemes—exercising through TR led to better health and structured exercise routine—and one hindering subtheme—lack of tailored exercise interventions—emerged to explain whether participants achieved health-related outcomes.

**1) Exercising through TR led to better health**

When the participants were asked about the reasons why they would continue to use TR, more than half of the participants gave the straightforward response that they believed that exercising would improve their health. They described the subjective feeling of improvements in their overall health after following the exercises on TR. Some even described functional improvements: for example, Participant 8 commented that “*even though the exercises were simple, I felt that my legs got better*… *I felt there was some effectiveness*”.

**2) Structured exercise routine**

Some participants likened the e-calendar function on the TR system to exercise homework that motivated them to complete it by the end of the day. They mentioned that by exercising daily, they got into the habit of exercising and were able to form their own exercise routines that they continued after the TR program had ceased. Participant 4 even commented that “*it’s not bad to share the exercises with others*”, explaining that she had demonstrated the exercises she had learned to those in her personal social circle.

**3) Lack of tailored exercise interventions**

When the participants were asked if there were any areas of improvement for TR, eight participants reported the lack of tailored exercise interventions to meet their health needs. Some participants reported that they felt that the exercises were too easy and that they had increased the number of repetitions on their own without consulting the therapists. On the contrary, others had stopped or reduced their exercises due to pain or difficult body positions encountered. Participant 2 queried whether Caspar Health had customized options, such as beginner, intermediate, and expert levels, and made the following comment about the program: “*it only had five exercises which I became very familiar with*… *seemed to be boring*…*no improvements*”. Other participants shared that the exercises were like foundational training and were not modified to meet the changes in their health status over the course of 6 weeks.

**Theme 3 - Other psychosocial and environmental factors that influenced the use of TR**

**1) Increased motivation to exercise through TR (psychosocial)**

Four participants highlighted that through the TR system, they had become more motivated to exercise and maintain their exercise routine through the e-calendar. Some shared that they were able to learn new exercises through the app and that this positive experience fed into their motivation to exercise post-intervention.

**2) Unmotivated to exercise (psychosocial)**

Conversely, three participants did not complete the prescribed exercises as they were “*lazy*” and “*lacked motivation*” to complete them. They also emphasized that the sole reason why they did not complete the exercises was their lazy habits; they did not attribute it to the use of TR and the iPhone. Participant 4 made the following comments: “*I do not exercise on a regular basis*… *it’s not that the iPhone is bad, maybe I am lazy, I will not do it every day*”.

**3) Environment**

Social support from family members was also highlighted as an important enabler in the use of TR in terms of troubleshooting the technical issues. Participant 12 commented on the support given by her daughter, stating “*she would demonstrate and teach me how to perform the prescribed exercises*”; this participant’s daughter was also able to resolve the technical difficulties her mother encountered.

While some participants reported that there was insufficient space within their residential dwellings to perform the exercises, others shared they would head outdoors to use TR app on the iPhones and exercise. Participant 6 reported that he possessed the flexibility “*to use it both indoors and outdoors*”.

***Theme 4 - Intention to use TR***

When asked whether they would continue to use the TR app, seven participants said that they would continue with its use and even recommend it to other patients. Some explained that it offered them the convenience to perform exercises at home and minimized social contact as the pandemic persists. Others specifically shared that they would choose TR so that healthcare resources were reserved and kept “*for others who need it*” (Participant 7). Moreover, Participant 2 suggested the use of a “*hybrid system*” as he felt that through a combination of onsite therapy sessions and TR sessions, therapists could modify the exercises to suit his condition and needs.

In contrast, four participants reported that they would not continue with nor recommend Caspar Health to others due to the variety of similar exercise-based apps in the market. Participants 5 and 11 shared that they would not recommend the app to others due to their unsatisfactory experiences, and both particularly commented that the app “*is very troublesome*”. Moreover, these participants stated that they would prefer to attend therapy sessions face-to-face as this does not require the charging of a mobile phone (Participant 3) and it would be easier to recall the exercises taught onsite (Participant 5).

## Discussion

The aim of this study was to explore the usability and acceptability of the TR system through the experiences of patients with long COVID in Hong Kong. While the usability of the TR system was reflected in the less than satisfactory SUS scores, most participants continued to perceive its use as marginally acceptable. The contrast between the usability and its perceived acceptability was exemplified through the interview responses that revealed despite the technical difficulties faced in its use, majority of participants were open to adopting the TR and would recommend it to others. These results differ from the increased uptake of TR and high patient satisfaction level in the observation study by Ku et al. (2021) where they tracked the use of TR when the pandemic disrupted hospital-based rehabilitation services in Hong Kong. Variations in results may stem from the differences in the built-in infrastructure and user interface of the TR systems which influence its ease of use and the likelihood of adoption by users. Also, as most participants gave a rating of 3 (Neutral) on the SUS items, as shown in Table 3, this could have indirectly introduced a response bias which contributed to the low mean SUS scores reported. However, through the qualitative data collection, this study uncovered the perceived enabling factors and barriers that influenced the participants’ intention to use the TR system.

Participants who were willing to continue TR and recommend it to others cited enabling factors such as the perceived improvements in their health, the psychological benefits of increased motivation to exercise, the ease of using TR, and the ease of following the prescribed exercise videos. These factors concur with the significant improvements in the physical functions of COVID-19 patients found in a systematic review conducted by Vieira et al. (2022) and the reduction in PACS. This is consistent to our previous review that both patients and caregivers were generally satisfied with the use of TR in providing occupational therapy services (Hung and Fong, 2019). Results from other studies further affirmed that perceived functional improvements encourage participants to continue rehabilitation through TR (Bernal-Utrera et al., 2021; Buabbas et al., 2022; Milani et al., 2021).

Moreover, it was understandable that the participants highlighted their preference to receive home-based TR in view of the uncertainties of lockdown and the ever-changing social distancing measures during the COVID-19 pandemic. Some participants appreciated the convenience of exercising at home and cited the psychological benefits of being more motivated to exercise. TR also offered the participants the flexibility to fit the exercise schedule into their daily routine. Hence, the exercise-based TR program could help patients to manage their persistent physical symptoms of COVID-19 and could also be used to circumvent the feelings of isolation, psychological distress, and anxiety faced while undergoing mandatory quarantine and social distancing (Chair et al., 2021; Zhao et al., 2020). As the smartphones were portable, they offered them the flexibility to perform TR outdoors and overcome the issue of restricted indoor space.

However, the lack of customized interventions to address the individual needs of participants suffered from PACS and the technology issues encountered in the use of the TR system were highlighted as the major barriers to the acceptance of TR in this study. Although participants shared that the TR program were beneficial, they were not tailored to their functional performances and led to unsatisfactory feelings of boredom; they also commented that it was arduous for them to use the system as reflected in the low SUS scores. Safety concerns were brought up: Without consulting the therapists, some participants increased the repetitions of the prescribed exercises, while others stopped performing the exercises altogether due to the pain they experienced. This might not be an innate issue with the TR interface but rather may be related to the lack of compliance with remote supervision from therapists. As most participants were first-time users of the TR App installed in iPhones, they were unfamiliar with operating them as compared to their usual smartphones and found it particularly hard to learn how to use them. This was highlighted in the experiences of participants 5 and 11, who gave the lowest SUS scores in relation to their TR use. This finding is consistent with that of a previous study using the Caspar Health TR system that similar problems occurred with some elderly patients using their own smartphones (Li et al., 2022). However, as this study did not examine other factors, such as their cognitive abilities and competency in using the app, we were unable to rule out whether these extraneous factors played significant roles in their difficulty they experienced using the TR system.

Moreover, their experiences were complicated by other personal factors, such as increased difficulty in learning and using new technology at an older age. The results of this study are consistent with the findings of Saito and Izawa (2021), who noted that older adults frequently encounter technology-related issues due to their unfamiliarity with novel technology and their aging-related difficulties. In their review, Pramuka and van Roosmalen (2009) also highlighted that TR systems that require steep learning curves or high levels of maintenance are at a higher risk of technology abandonment. However, these issues could be addressed with support from family members, as pointed out by Participant 12, whose daughter assisted her to troubleshoot the technical issues she faced. It was suggested in a paper by Chung et al. (2021) that older adults who receive technology support from their caregivers have a higher likelihood of using TR. Therefore, caregivers could also be trained in the use of TR to assist participants; they could even provide onsite supervision to reduce the safety risks associated with TR (Vieira et al., 2022). In addition, therapists could conduct regular phone follow-ups with patients to ensure their training needs are met, address the troubleshoot issues faced with their individual regime and encourage their adherence to the prescribed TR program (Fioratti et al., 2021). Routine phone check-ins might also detect other issues, like the lack of tailored interventions among the participants, earlier on and address them with updated customized interventions.

This study has some limitations. Although this study adopted a mixed-methods design and previous usability studies on TR also show that a small sample is appropriate to obtain qualitative feedbacks from participants (Toh et al., 2023), a larger sample size may improve the external validity of the findings to other patients with PACS. Additionally, it increases susceptibility to random variations, as reflected by the skewed median results, and constrains the ability to explore the impact of gender differences. As the phone interviews were conducted 6 months after the TR interventions had been completed, selection and recall biases might have resulted in missing and skewed results (Talari and Goyal, 2020). As iPhones were loaned to the participants, they might not have had access to TR once the technology infrastructure was returned to the therapist after the course of training. As many of the participants were using an iPhone for the first time, this might have resulted in biased responses due to their unfamiliarity with the system. In addition, as all of the participants received a specific duration of TR, the results cannot be generalized to all patients who have had COVID-19. Unfortunately, as this study did not have access to the participants’ compliance rate with the TR program prescribed on the Caspar Health app, the results of this study could not be triangulated further with their actual use of the TR app. Lastly, as telephone interviews were conducted due to the pandemic situation, this might have reduced opportunities for the researchers to observe non-verbal cues and conduct face-to-face social interactions.

Therapists are the driving force for the uptake of TR as they can prescribe and promote its use among their patients (Intan Sabrina and Defi, 2021). Without their prescription and use of TR, the result may be the abandonment of rehabilitation technology (Pramuka and van Roosmalen, 2009). In addition, therapists play an important role as facilitators to ensure patients’ compliance with the prescribed TR program through remote monitoring and close follow-up (Fioratti et al., 2021). Hence, future studies could consider comparing the perspectives of therapists against those of end-users to generate a wider view of the facilitators of and barriers to TR (Blanco et al., 2022; Ganesan et al., 2021).

Results from a previous study (Cranen et al., 2012) highlighted that even though TR was valued by the participants, they were still hesitant to use it as their sole treatment method. As suggested by Participant 2, a hybrid delivery model that consists of both in-person care and virtual TR sessions could be explored to determine if it can promote the benefits from both modes of rehabilitation. Hybrid delivery approach may reduce the overall costs of the sessions, but increase the accessibility of rehabilitation to participants’ homes despite social distancing measures, and maintain therapeutic interactions between therapists and patients during onsite sessions (Ganesan et al., 2021; Raj Westwood, 2021; Wong et al., 2022). Future studies should also consider different TR models and systems using the hybrid delivery approach and triangulate them against the perceived acceptability of TR system to determine its clinical utility. Also, as PACS is considered a multifaceted chronic condition with heterogeneous symptom manifestations, it may be noteworthy to explore the use of various treatments by an interdisciplinary team to get patients the best care in future.

## Conclusion

The results from this mixed-methods study demonstrate that participants with long COVID were generally satisfied with their use of the occupational therapy TR system during the pandemic in addressing their healthcare needs. The perceived health and psychosocial benefits from the use of TR, its ease of use, and having social support played roles as facilitators to encourage the use of TR among the participants. However, barriers such as technical difficulties, the lack of customized exercises, and the difficulty of using novel mobile technology at an older age might have resulted in the fair usability perception of the TR. Despite this, participants still recognized the potential benefits of the TR system and expressed willingness to learn and adopt it, as reflected in their acceptability scores and interviews. Future research could consider exploring the use of various TR models such as the hybrid delivery approach for patients who have reservations about the use of TR alone, thus allowing them to experience the perceived benefits reported in this study.

## Data Availability

The datasets presented in this article are not readily available because consent for using the data had not been approved by participants. Requests to access the datasets should be directed to KF, rsnkfong@polyu.edu.hk.

## Footnotes

1
https://apps.apple.com/hk/app/caspar-health/id1222630969

## Appendix

**Appendix:** Semi-structured questionnaire.

Topic	Guiding questions
Introduction	Do you currently undergo rehabilitation services? If yes, what type of rehabilitation services?
General experiences	For the next portion, I would like you to think about the time when you participated in the CASPAR telerehabilitation study and the researchers had given you some exercises and relaxation videos to watch on the iPhone: Did you follow the exercise and education videos given by the therapists on CASPAR?
	How was your experience of using CASPAR at home?
	What did you like about CASPAR?
	What did you dislike about CASPAR?
Usability	How did you find using the CASPAR application on the iPhone? Was it easy or difficult to use it?
	Were there components in CASPAR that you think can be improved on?
	Did you need help when using CASPAR? Which part did you need help with?
	If given the option to continue or receive rehabilitation through CASPAR, would you continue to use it? If yes, why? If not, why?
	In response to the current pandemic situation, if there are some other modes of service to replace the current face-to-face rehabilitation training temporarily, which training would you choose? (e.g., home education program from therapists, home therapy, telerehabilitation CASPAR?) and why?
	Would you recommend CASPAR for others to use?
Conclusion	Is there anything else that you would like to add regarding the topic?
